# Composition rules of Ni-base single crystal superalloys and its influence on creep properties via a cluster formula approach

**DOI:** 10.1038/s41598-020-78690-8

**Published:** 2020-12-10

**Authors:** Chen Chen, Qing Wang, Chuang Dong, Yu Zhang, Honggang Dong

**Affiliations:** 1 Key Laboratory of Materials Modification by Laser, Ion and Electron Beams (Ministry of Education), School of Materials Science and Engineering, Dalian University of Technology, Dalian, 116024 China; 2 Department of Basic Courses, Liaoning Institute of Science and Technology, Benxi, 117004 China

**Keywords:** Engineering, Materials science

## Abstract

The present work investigated the composition evolution of the TMS series of Ni-base single crystal (SC) superalloys in light of the cluster formula approach systematically. The cluster formula of SC superalloys could be expressed with ${[}\overline{{{\text{Al}}}} {-} \overline{{{\text{Ni}}}} 12{](}\overline{{{\text{Al}}}} {, }\overline{{{\text{Cr}}}} {)}m$, in which all the alloying elements were classified into three groups, Al series ($\overline{{{\text{Al}}}}$), Cr series ($\overline{{{\text{Cr}}}}$), and Ni series ($\overline{{{\text{Ni}}}}$). It was found that the total atom number (*Z*) of the cluster formula units for TMS series of superalloys varies from *Z* ~ 17 to *Z* ~ 15.5, and then to *Z* ~ 16 with the alloy development from the 1st to the 6th generation, in which the superalloys with prominent creep resistance possess an ideal cluster formula of ${[}\overline{{{\text{Al}}}} {-} \overline{{{\text{Ni}}}} 12{](}\overline{{{\text{Al}}}} 1.5\overline{{{\text{Cr}}}} 1.5{)}$ with *Z* = 16. Similar tendency of composition evolution also appears in the PWA and CMSX series of SC superalloys. Typical TMS series of superalloys with prominent creep properties generally exhibit a moderate lattice misfit of γ/γ′ which could render alloys with appropriate particle size of cuboidal γ′ precipitates to acquire a maximum strength increment by precipitation strengthening mechanism. More importantly, the relationship between the lattice misfit (*δ*) of γ/γ′ and the creep rupture lifetime (*t*_*r*_) of superalloys was then established, showing a linear correlation in the form of lg*t*_*r*_–lg|*δ*|^3/2^ at both conditions of 900 °C/392 MPa and 1100 °C/137 MPa. Combined with the lattice misfit, the cluster formula approach would provide a new way to modify or optimize the compositions of Ni-base superalloys for further improvement of creep property.

## Introduction

The prominent high-temperature (HT) mechanical properties of Ni-base single crystal (SC) superalloys are primarily benefited from their unique coherent microstructure with cuboidal ordered L1_2_-γ′ (Cu_3_Au-type) nanoparticles precipitated in the face-centered-cubic (FCC) γ solid solution matrix^1^. This kind of coherent microstructure strengthened by ordered phase is closely related to the lattice misfit (*δ*) between γ and γ′ phases that depends on their chemical compositions. Actually, not only the morphology (particle shape and size) and distribution of nanoprecipitates, but also the resistance to HT creep of superalloys rely on the lattice misfit^2–7^. Thereof, a dozen of alloying elements were generally added into the Ni base matrix and the amounts of these elements were controlled precisely to achieve a moderate lattice misfit for the improvement of the microstructural stability and the rupture strength/lifetime of alloys^8–11^. For instance, the rupture lifetimes of TMS-138 (Ni–5.8Co–5.9Al–5.6Ta–3.2Cr–2.8Mo–5.9W–5Re–2Ru, weight percent wt%) and TMS-75(+Ru) (Ni–12Co–6Al–6Ta–3Cr–2Mo–6W–5Re–1.6Ru wt%) SC alloys are *t*_*r*_ = 412 h and *t*_*r*_ = 143 h, respectively, in the condition of a temperature of 1100 °C and an applied stress of 137 MPa^12,13^. The difference in rupture lifetime results from that the lattice misfit (*δ* = − 0.33%) of γ/γ′ in TMS-138 is negatively larger than that (*δ* = − 0.16%) in TMS-75(+Ru), which renders TMS-138 with a much higher interfacial strength between γ/γ′ to prevent dislocations from cutting into the γ′ particles and to form high-density dislocation networks on γ/γ′ interfaces.

Up to now, six generations of TMS series of Ni-base SC superalloys have been developed to improve gradually their comprehensive properties at HTs, during which the amounts of alloying elements were finely tuned to realize an optimization of alloy properties^14–22^. In order to enhance the oxidation-resistance, a small amount of Si element (< 0.5 wt%) could be added into alloys to form compact oxide scales combined with the contribution of Al element^23–26^. However, excessive Si is detrimental to the mechanical property since it could accelerate the precipitation of topological close packed (TCP) phases^24,27^. In addition, the addition of Mo element into the TMS-6 SC alloy (Ni–5.3Al–9.2Cr–10.4Ta–8.7W wt%) could improve the thermomechanical fatigue property of alloys, in which the optimum reaches only at an appropriate amount of Mo (~ 0.6 wt%). Actually, all these improvements are at the expense of the creep rupture lifetime of superalloys, as demonstrated by the fact that the rupture lifetime of TMS-6 at 1100 °C/137 MPa decreases from *t*_*r*_ = 340 h to *t*_*r*_ = 236 h with the addition of 0.6 wt% Mo, and to *t*_*r*_ = 164 h with 1.2 wt% Mo^23^. Thus, the precise control of alloy composition is of great importance to achieve an optimal comprehensive property, which is difficult to realize due to the composition complexity.

Several computation-aided design methods have been applied into the development of Ni-base SC superalloys, such as PHACOMP (a phase computation method based on the electron vacancy concept)^28–30^, New PHACOMP based on the *d*-electrons theory^31–34^, ADP (an alloy design program based on a mathematical model using regression equations in microstructure and property databases)^14,35^, Multi-criteria Numerical Optimization (an improved numerical multi-criteria optimization tool using CALPHAD calculations and semi-empirical models)^36,37^, Alloys-by-Design (a design model by incorporating the complex interactions among alloy composition, microstructure and processing)^38,39^, and so on. Among them, the classical New PHACOMP was well developed by calculating the electronic structure of alloys with the DV-Xα molecular orbital method, in which the important parameter $\overline{{M{\text{d}}}}$ represents an average energy level of *d* orbitals of transition metals and is used to predict the phase stability of FCC-γ matrix and the precipitation of TCP phase^31–34^.

In our previous work, we used a cluster formula approach to explore the composition rules of Ni-base superalloys, in which all alloying elements are classified into three groups, Al-like $\overline{{{\text{Al}}}}$ series, Cr-like $\overline{{{\text{Cr}}}}$ series, and Ni-like $\overline{{{\text{Ni}}}}$ series, according to the interaction between the alloying element with the base Ni^40,41^. Thus, a uniform cluster formula of ${[}\overline{{{\text{Al}}}} {-} \overline{{{\text{Ni}}}} 12{](}\overline{{{\text{Al}}}} {, }\overline{{{\text{Cr}}}} {)}m$ was obtained to analyze alloy composition, which would be described in details in the next section. Actually, the composition evolution of superalloys was not distinguished when multi-target properties were considered simultaneously, which was ascribed to an obvious trade-off between the creep property and the oxidation- and corrosion-resistances. Therefore, the present work will focus on the TMS series of SC superalloys (1st–6th generations) to explore the composition rules of alloy development in light of the cluster formula approach. Based on it, the relationship between the lattice misfit of γ/γ′ and the creep rupture lifetime of SC superalloys will be established, in which the influence of lattice misfit on the strengthening by γ′ precipitates is also discussed. In addition, the composition variation of TMS series of superalloys will be also explored when other properties (e.g. oxidation- and corrosion-resistances, etc.) are considered.

## Composition rules of Ni-base SC superalloys via the cluster formula approach

### Cluster formula approach

The cluster-plus-glue-atom model^42,43^ was used to describe the local atomic distribution of alloying elements in solid solutions based on the chemical short-range orders (CSROs) proposed initially by Colley^44^ and Friedel^45^. CSROs are the most typical structural characteristics of solid solutions and represent the local structural heterogeneities caused by the distribution of solute atoms, which plays an important role in various mechanical and physical properties of alloys^46–48^. The strongest CSRO is generally incarnated into the nearest-neighbor cluster centered by a solute atom that has a strong interaction with the base solvent atom. Some other solute atoms exhibiting a relatively weak interaction with the base would fill into the glue atom sites among clusters to balance the atomic-packing density^49^. Thus, this cluster model can visualize the CSRO as a local structural unit consisting of a nearest-neighbor cluster surrounded by several glue atoms. A composition formula [cluster](glue atom)_*m*_ (*m* being the glue atom number) can be then obtained from the cluster structural unit, in which the nearest-neighbor clusters are cuboctahedron with a coordination number of 12 (CN12) and rhombi-dodecahedron with CN14 in FCC and BCC solid solutions, respectively^42,43^.

It has been demonstrated by neutron experiments that the local atomic distributions of solute elements in solid solutions are in a good consistence with those in their related ordered phase, which is closely related to the content of solute elements^50–52^. We have classified the alloying elements into the $\overline{{{\text{Al}}}}$, $\overline{{{\text{Cr}}}}$, and $\overline{{{\text{Ni}}}}$ series, respectively, and the sites of these elements in the cluster formula are determined by the interaction between the solute elements and the base Ni (characterized by the enthalpies of mixing (*ΔH*)^40,53^). Since the $\overline{{{\text{Al}}}}$ series of elements (Al, Nb, Ta, Ti, and Si) have large negative *ΔH* with the base Ni (*ΔH*_Al–Ni_ = − 22 kJ/mol, *ΔH*_Nb–Ni_ = − 30 kJ/mol, *ΔH*_Ta–Ni_ = − 29 kJ/mol, *ΔH*_Ti–Ni_ = − 35 kJ/mol and *ΔH*_Si–Ni_ = − 40 kJ/mol^53^), they would like to be in the cluster center preferentially to form such a ${[}\overline{{{\text{Al}}}} {-}\overline{{{\text{Ni}}}} 12{]}$ cluster in FCC solid solution and the excessive amount of elements could also enter into the glue atom sites. By contrast, the $\overline{{{\text{Cr}}}}$ series of elements, including Cr, Mo, and W, tend to occupy the glue atom sites due to the relatively weaker *ΔH* with Ni (*ΔH*_Cr–Ni_ = *ΔH*_Mo–Ni_ = − 7 kJ/mol and *ΔH*_W–Ni_ = − 3 kJ/mol). And the $\overline{{{\text{Ni}}}}$ series of elements (Co, Re, Ru, and Ir) would occupy the cluster shell to substitute for the base Ni due to that their *ΔH* values are nearly zero^53^. Thus, the compositions of Ni-base SC superalloys could be expressed with the cluster formula of ${[}\overline{{{\text{Al}}}} {-}\overline{{{\text{Ni}}}} 12{](}\overline{{{\text{Al}}}} {, }\overline{{{\text{Cr}}}} {)}m$, in which the total atom number (*Z*) of the cluster formula unit is equal to *Z* = 13 + *m*. Since the glue atoms in the cluster structural model are used to fill in the interstitial sites of cluster packing for the balance of the atomic density, the glue atom number *m* and the total atom number *Z* could be calculated in light of the Friedel oscillation theory when the cluster is fixed^49^. It has been demonstrated that the calculated values are *Z* = 16 and *m* = 3 with the CN12 cluster for both multi-component Ti alloys and Cu alloys^49,54^. A similar calculation in simple Ni–Cr–Al ternary system also indicates that the ideal values are *Z* = 16.16 and *m* = 3.16, respectively, close to the ideal integer value of *Z* = 16 (*m* = 3) in the cluster formula [Al-Ni_12_](Al,Cr)_*m*_^55^.

### Composition rules of TMS series of SC superalloys via the cluster formula approach

Typical alloy compositions of TMS series of Ni-base SC superalloys from the 1st generation to the 6th generation, including both the weight percent (wt%) and the atomic percent (at.%), are listed in Table S1 (Supplementary Materials). According to the ratio of the atomic percent to the atom number of 12 of $\overline{{{\text{Ni}}}}$ series of elements in the cluster formula, the atom number of each element in the cluster formula unit could be calculated with the Eq. (1):

1 $$\begin{aligned} n_{i} & = 12 \times x_{i} /x_{{\overline{{{\text{Ni}}}} }} \\ Z & = 12 + \sum\limits_{i} {n_{i} = 1 + 12 + m} \\ \end{aligned}$$

where *n*_*i*_ and *x*_*i*_ are the atom number in the cluster formula unit and atomic percent of each element *i* in the $\overline{{{\text{Al}}}}$ and $\overline{{{\text{Cr}}}}$ series, respectively; $x_{{\overline{{{\text{Ni}}}} }}$ is the total atomic percent of the $\overline{{{\text{Ni}}}}$ series of elements; and *Z* and *m* are the total atom number and the glue atom number of the cluster formula unit, respectively. In addition, the atom number of each element in the $\overline{{{\text{Ni}}}}$ series could be obtained according to their atomic percent ratio in a total atom number of 12. Thus, the cluster formulas of all these TMS series of superalloys were analyzed and listed in Table S1, including the *Z* value, the glue atom number *m*, as well as the atom number of $\overline{{{\text{Cr}}}}$ series ($G_{{\overline{{{\text{Cr}}}} }}$) and $\overline{{{\text{Al}}}}$ series ($G_{{\overline{{{\text{Al}}}} }}$) in glue atoms, i.e., $G_{{\overline{{{\text{Al}}}} }} = m - G_{{\overline{{{\text{Cr}}}} }}$. We will re-consider the composition evolution of the TMS series of superalloys in light of the cluster formula approach.

Table S1 also lists the creep rupture lifetimes *t*_*r*_ in both conditions of 900 °C/392 MPa and 1100 °C/137 MPa of these SC superalloys, from which it could be found that the composition evolution of SC alloys is closely related to the creep rupture lifetime, exhibiting a gradually enhanced tendency from the 1st to the 6th generation. For example, the creep rupture lifetime of the TMS-6 (1st generation SC superalloy) at 1100 °C/137 MPa is *t*_*r*_ = 340 h^23^, and it could be improved up to *t*_r_ = 722 h in TMS-138A (4th generation)^56^, and then to *t*_r_ = 1001 h in TMS-196 (5th generation)^12^ and to *t*_r_ = 1930 h in TMS-238 (6th generation)^21^ through fine-tuning the amounts of alloying elements. In addition, some superalloys have been developed from a specific SC superalloy, as demonstrated by the fact that both TMS-138A-Cr + Si and TMS-138A-Mo + Si alloys were come from the TMS-138A in order to improve their HT corrosion- and oxidation-resistances at the cost of rupture lifetime^24^. While the TMS-138A was derived from the original TMS-138 for the further improvement of rupture lifetime.

**Figure 1:**
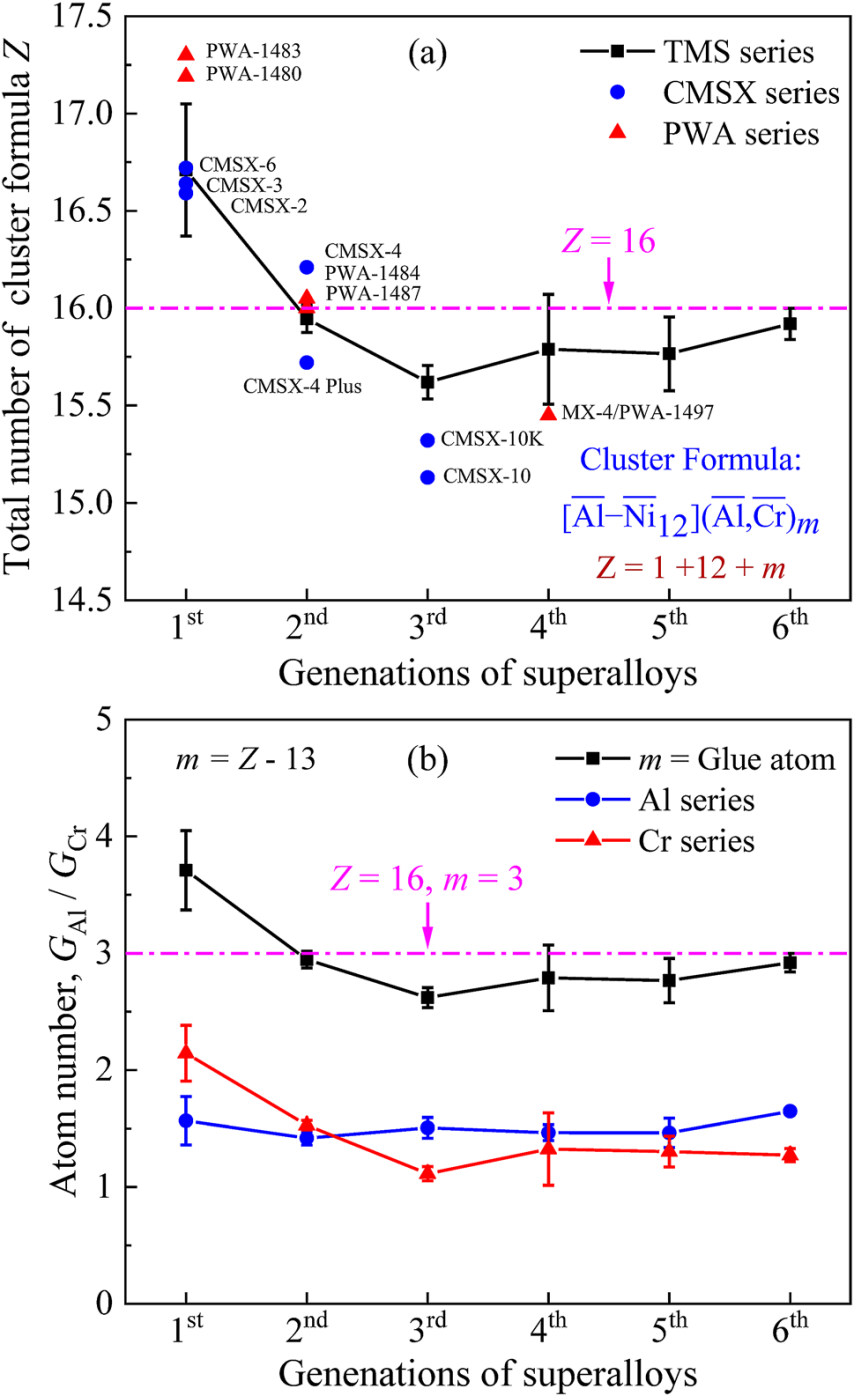
Variation of atom numbers in cluster formula with the developed generations of Ni-base SC superalloys. (**a**) The total atom number *Z* of the cluster formula; (**b**) the atom number of the $\overline{{{\text{Al}}}}$ series, $\overline{{{\text{Cr}}}}$ series at the glue atom sites, and the glue atom number.

From the viewpoint of cluster formulas of these six generations of TMS SC superalloys, the variations of the total atom number *Z* in the cluster formula unit with the generations of these alloys were investigated, as seen in Fig. 1a. The *Z* value decreases from *Z* ~ 17 to *Z* ~ 15.5 with the generation increasing from the 1st to the 3rd one, and then increases to *Z* ~ 15.8 with the generation from the 3rd one to the 6th. Finally, the *Z* value will tend to be *Z* = 16, which is also the total atom number of the cluster unit in the 2nd generation of TMS superalloys (*Z* = 15.8–16.0). Moreover, another two kinds of SC superalloys, the CMSX and PWA series, were also investigated via the cluster formula approach. It is found that the *Z* values of these two series of alloys also follow the same tendency of the TMS series (Fig. 1a), as exampled by the *Z* = 16.0 for both the PWA1484 and PWA1487 superalloys (the 2nd generation of PWA series).

For the $\overline{{{\text{Al}}}}$ series of elements, the total amounts in these TMS series of SC superalloys are almost constant, exhibiting a total atom number in the cluster formula of $1 + G_{{\overline{{{\text{Al}}}} }} = 2.5$, as seen in Fig. 1b. The primary role of the $\overline{{{\text{Al}}}}$ series of elements is to form the γ′ nanoprecipitation and to keep the volume fraction of γ′ precipitates at a high level of about 50–70%^57,58^. On the contrary, the total amount of $\overline{{{\text{Cr}}}}$ series of elements, expressed with the atom number $G_{{\overline{{{\text{Cr}}}} }}$ in the cluster formula, varies drastically from $G_{{\overline{{{\text{Cr}}}} }}$ = 2.4 in the 1st generation to $G_{{\overline{{{\text{Cr}}}} }}$ = 1.2 in the 6th generation. Such similar variation tendency also appears in the glue number *m* (*m* = *Z* − 13) of the cluster formula unit (Fig. 1b). Especially, the Cr content decreases gradually, as evidenced by the fact that it varies from 9 wt% in the 1st generation of SC superalloys to about 5 wt% in the 2nd generation, then to 2–3 wt% in the 3rd–5th generations. This reduction of Cr might be considered to inhibit the formation of brittle TCP phase (Cr/Re-rich) due to the gradual increase of Re in superalloys from the 1st to the 5th generation^59–61^. Besides, in the latest 6th TMS-238 alloy, the Cr content increases again to about 5 wt%, similar to that in the 2nd generation of superalloys. In fact, the adjustment of the amount of $\overline{{{\text{Cr}}}}$ series is mainly attributed to achieve a moderate lattice misfit between γ and γ′ phases for an optimal creep-resistant property, which is expressed with the formula of *δ* = 2 (*a*_γ′_ − *a*_γ_)/(*a*_γ′_ + *a*_γ_) (*a*_γ_ and *a*_γ′_ being the lattice constant of γ and γ′ phases, respectively). The lattice misfit values of these TMS series of superalloys at both 900 °C and 1100 °C are also listed in Table S1, together with the rupture lifetime, which will be discussed in the following. It is noted that the currently-cited *δ* values at 900 °C were all calculated by the NIMS in-house alloy design program (NIMS-ADP), and those at 1100 °C were mainly obtained by the HT XRD measurements.

In the $\overline{{{\text{Ni}}}}$ series of elements, the addition of a small amount of Re and Ru can further modulate the γ/γ′ lattice misfit for the improvement of the creep properties^62,63^. However, it should be noted that the excessive addition of Re would accelerate the precipitation of detrimental TCP phases^59,60^. Since the Ru addition could counteract the microstructural instability caused by high content Re, the co-addition of Re and Ru with an appropriate amount would like to be carried on in the late 4th–6th generations of SC superalloys.

Therefore, all the $\overline{{{\text{Al}}}}$ series, $\overline{{{\text{Cr}}}}$ series, and $\overline{{{\text{Ni}}}}$ series of elements should be tuned simultaneously to balance the HT mechanical property and the oxidation/corrosion resistances. Consequently, the optimum comprehensive property will reach at the superalloy with the cluster formula of ${[}\overline{{{\text{Al}}}} {-}\overline{{{\text{Ni}}}} 12{](}\overline{{{\text{Al}}}} 1.5\overline{{{\text{Cr}}}} 1.5{)}$ (*m* = 3), which is well consistent with the ideal cluster formula with the atom number of *Z* = 16 in diverse alloy systems^40,49,54,55^.

## Correlations among lattice misfit, particle morphology, and creep property

### Precipitation strengthening mechanism

It is well-known that the morphology of precipitates, including the particle shape and size, as well as the volume fraction are crucial to the mechanical properties of alloys, especially the HT creep property^58,64,65^. For the precipitation strengthening, the strengthening mechanism can be divided into two categories, the Orowan bowing mechanism and the particle shearing mechanism, depending on the interaction between moving dislocations and precipitates. The Orowan mechanism generally occurs when the particles are large or incoherent with the matrix, while the shearing mechanism dominates when the precipitate are coherent and small. For the shearing mechanism, three factors contribute to the increment in yield strength, coherency strengthening (*Δσ*_*CS*_), modulus mismatch strengthening (*Δσ*_*MS*_), and order strengthening (*Δσ*_*OS*_). Among them, the former two (*Δσ*_*CS*_ and *Δσ*_*MS*_) occur prior to the shearing of precipitates by dislocations, and the latter one (*Δσ*_*OS*_) occurs during shearing. Thus, the larger value of (*Δσ*_*CS*_ + *Δσ*_*MS*_) or *Δσ*_*OS*_ would be responsible to the strength increment (*Δσ*_*shearing*_) from the shearing mechanism. Since, the Orowan mechanism and particle shearing occur concurrently and are independent to each other, the final strengthening increment is determined by the smaller value of *Δσ*_*shearing*_ or *Δσ*_*orowan*_. The equations available to calculate strength increments are expressed with Eqs. (2–5)^64–69^:

2 $$\Delta \sigma_{CS} = M \times a_{\varepsilon } \times \left( {G\delta_{c} } \right)^{3/2} \times \left( {rf/0.5Gb} \right)^{1/2}$$

3 $$\Delta \sigma_{MS} = M \times 0.0055 \times \left( {\Delta G} \right)^{3/2} \times \left( {2f/G} \right)^{1/2} \times \left( {r/b} \right)^{3m/2 - 1}$$

4 $$\Delta \sigma_{OS} = M \times 0.81 \times \left( {\gamma_{{{\text{APB}}}} /2b} \right) \times \left( {3\pi f/8} \right)^{1/2}$$

5 $$\begin{aligned} \Delta \sigma_{{{\text{orowan}}}} & = M \times \left( {0.4Gb/\pi \sqrt {1 - v} } \right) \times \left( {\ln \left( {2\sqrt {2/3} r/b} \right)/\lambda_{p} } \right) \\ \lambda_{p} & = 2\sqrt {2/3} r\left( {\sqrt {\pi /4f} - 1} \right) \\ \end{aligned}$$

where *M* = 3.06 for FCC structure (Taylor factor)^68^, *α*_*ε*_ = 2.6 (a constant)^66,67^, *m* = 0.85 (a constant)^70,71^, *δ*_*c*_ = 2*δ*/3^66,67^, the constrained lattice misfit. *G* = 81 GPa and *ΔG* = 4 GPa are the shear modulus of the matrix and the shear modulus misfit between precipitates and matrix, respectively^72^; *b* = 0.254 nm is the Burgers vector^6,72^; *r* is the average particle size (i.e., the average edge length of cuboidal precipitates measured from experimental observations) and *f* is the volume fraction of the γ′ precipitates, respectively; γ_APB_ = 0.12 J/m^2^ is the anti-phase boundary energy of γ′-Ni_3_Al^72^; *ν* is the Poisson ratio (*ν* = 0.35 for Ni-base superalloys^73^), and *λ*_*p*_ is the inter-precipitate space.

**Figure 2:**
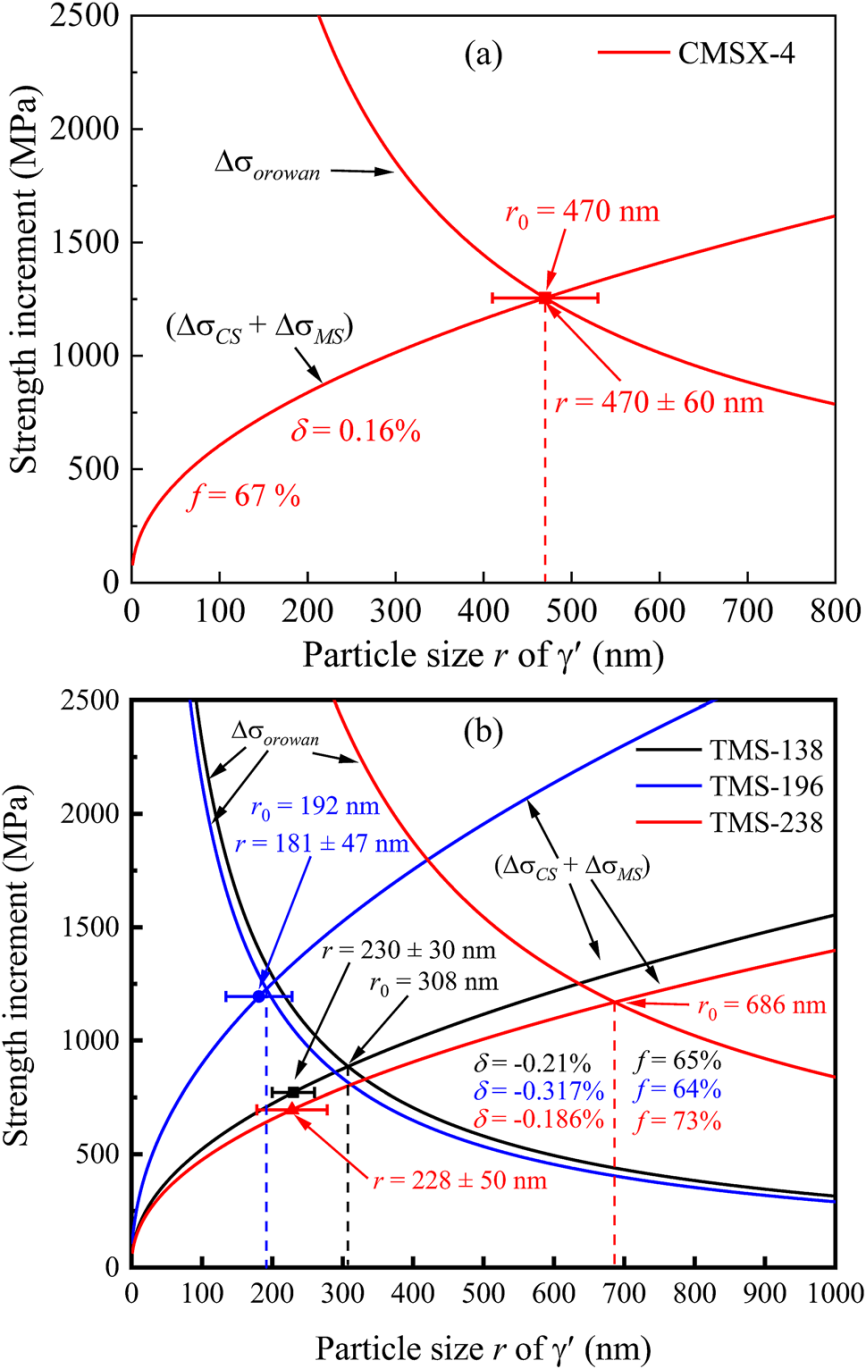
Computations of (Δσ_*CS*_ + Δσ_*MS*_) and Δσ_*orowan*_ as a function of particle size *r* of γ′ for the CMSX-4 (**a**) and TMS-138, TMS-196, and TMS-238 superalloys (**b**), in which the optimal particles sizes (*r*_0_) and the experimentally-measured *r* values, as well as the volume fraction *f* of γ′ precipitates are also marked for each alloy.

Based on these equations, it is addressed that the strength increment from the order strengthening (*Δσ*_*OS*_) of these series of superalloys are much smaller than that from coherency strengthening and modulus mismatch (*Δσ*_*CS*_ and *Δσ*_*MS*_) due to that these γ′ precipitates are large enough with a particle size of about 200–600 nm. For instance, the lattice misfit between γ and γ′ in CMSX-4 SC superalloy is *δ* = 0.16% at room temperature, and the particle size and the volume fraction of γ′ precipitates are *r* = 470 ± 60 nm and *f* = 67 ± 3%, respectively^74^. Thus, the strengthening increment from the total increment of the coherency strengthening and modulus mismatch in CMSX-4 is *Δσ*_*CS*_ + *Δσ*_*MS*_ = 1255 MPa, much larger than the strength increment from the order strengthening (*Δσ*_*OS*_ = 520 MPa). Thereof, the strength increment from shearing is determined by the value of (*Δσ*_*CS*_ + *Δσ*_*MS*_). It is worth mentioning that the strengthening effect caused by (*Δσ*_*CS*_ + *Δσ*_*MS*_) or *Δσ*_*orowan*_ is closely related to the lattice misfit *δ*, particle size *r*, and volume fraction *f* of precipitates, especially the *δ*. If the volume fraction *f* is fixed, the largest yield strength increment will be reached when (*Δσ*_*CS*_ + *Δσ*_*MS*_) = *Δσ*_*orowan*_ at an optimal particle size *r*_0_. Figure 2a plots the strength increment of (*Δσ*_*CS*_ + *Δσ*_*MS*_) or *Δσ*_*orowan*_ as a function of *r* of the CMSX-4 superalloy, in which the optimal size *r*_0_ value was calculated as *r*_0_ = 470 nm. Intriguingly, the theoretically calculated particle size is in a good agreement with the experimentally-measured value, which indicates that the maximum yield strength could be achieved experimentally.

Furthermore, the strength increments of 4th TMS-138, 5th TMS-196, and 6th TMS-238 superalloys were also calculated by using their *δ* and *f* parameters measured at 900 °C, where *G* = 47.4 GPa and *ΔG* = 3 GPa^6,75^. It is noted that the coarsening of γ′ precipitates is not obvious at this temperature, and these precipitates are still in a cuboidal shape and the precipitate size is comparable to that at room temperature^6,58^. The strength variations with the particle size are shown in Fig. 2b, in which the experimental particle sizes of these alloys are also marked. It could be found that the experimental particle size (*r* = 181 ± 47 nm) of TMS-196 alloy is well consistent with the calculated optimal value (*r*_*0*_ = 192 nm) with the volume fraction of *f* = 64%^76^, resulting in a maximum strength increment, compared with the other two alloys. Meanwhile, the 5th TMS-196 exhibited a prominent creep property, as demonstrated by the fact that the creep rupture lifetime (*t*_*r*_ = 1245 h) at 900 °C of this alloy is higher than that (*t*_*r*_ = 987 h) of 4th TMS-138 obviously^77,78^. It is primarily ascribed to the lattice misfit of γ/γ′, being *δ* = − 0.317% in the former and *δ* = − 0.21% in the latter, respectively, since their volume fractions are comparable (Fig. 2b). Generally, a relatively larger lattice misfit could produce a much higher strength and a much stronger γ/γ′ interface to prevent the dislocations from cutting γ′ precipitates, which would decrease the creep strain rate and thus improve the rupture lifetime. However, an exaggerated lattice misfit would accelerate the coarsening and rafting of γ′ precipitates, resulting in a reduction of rupture life.

Although the 6th TMS-238 alloy has a relatively lower lattice misfit (*δ* = − 0.186%) compared with the 5th TMS-196 and the 4th TMS-138, it still possesses the highest rupture lifetime (*t*_r_ = 1306 h) at 900 °C among these three alloys^79^, which is contributed to the relatively higher volume fraction (*f* = 73%). The higher volume fraction renders the TMS-238 alloy with a much higher strength increment contributed by the Orowan mechanism, as seen in Fig. 2b, which would produce a large calculated optimal particle size at *r*_*0*_ = 686 nm, much larger than the experimentally measured value (*r* = 228 ± 50 nm)^22^. This indicates that the coherent strengthening would be still dominant and be enhanced during the particle coarsening, which is different from the Orowan strengthening in the other two alloys. Thereof, the increase of volume fraction of γ′ precipitates is another approach to enhance alloy strength, since the γ channels would become narrow at a higher volume fraction to hinder the movement of dislocations.

It is noted the total atom number *Z* values in the cluster formula units of TMS-138, TMS-196, and TMS-238 superalloys are 15.63, 15.80, and 15.84, respectively, which are all close to the ideal value of *Z* = 16, especially for the latter two superalloys. Although the *Z* of TMS-196 (Ni–5.6Al–5.6Co–4.6Cr–0.1Hf–2.4Mo–6.4Re–5Ru–5.6Ta–5W wt%) is almost equal to that of TMS-238 (Ni–5.9Al–6.5Co–4.6Cr–0.1Hf–1.1Mo–6.4Re–5Ru–7.6Ta–4W wt%), the atom numbers of $\overline{{{\text{Al}}}}$ series (Al, Ta) and $\overline{{{\text{Cr}}}}$ series (Cr, Mo, W) of elements are different, being 2.39 and 1.41 for TMS-196, and 2.62 and 1.22 for TMS-238, respectively, where the amounts of Re and Ru in $\overline{{{\text{Ni}}}}$ series are constant. It is the fine tuning of the amounts of $\overline{{{\text{Al}}}}$ and $\overline{{{\text{Cr}}}}$ series of elements that changes the lattice misfit of γ/γ′ and then the maximum strength and its corresponding particle size. Therefore, it is crucial to consciously control the matching of alloying elements within the frame of the cluster formula unit with *Z* = 16, which could achieve a moderate lattice misfit of γ/γ′ for the optimization of mechanical properties of superalloys.

### Correlation between the lattice misfit and creep rupture lifetime

During the creeping process in either condition of an intermediate-temperature (900 °C)/intermediate-stress (392 MPa) or a high-temperature (1100 °C)/low-stress (137 MPa), it is emphasized that lattice misfit of γ/γ′ could strongly affect the microstructural evolutions of SC superalloys, including the initial γ′ particle size, the formation of high-density dislocation networks at γ/γ′ interfaces, and the coarsening and rafting of γ′ particles, which will result in various rupture lifetimes^6,62,80^.

Firstly, the particle morphology of γ′ is closely related to the lattice misfit of γ/γ′. As the above mentioned, an increase in lattice misfit could not only augment the particle size of γ′ precipitates, but also change the particle shape from spheroid to cuboid, and then to other irregular shapes^3,81^. Thereof, a moderate negative lattice misfit could obtain an optimal particle size and uniform distribution of cuboidal γ′ precipitates at the initial state, which would lead to a perfect continuous lamellar microstructure at HT to prevent dislocations from climbing during the creep process. Secondly, the lattice misfit is also of great importance to the strength of γ/γ′ interface. A higher magnitude of lattice mismatch could result in a finer spacing of misfit dislocations and a stronger γ/γ′ interface, which would in turn act as a stronger barrier to the moving dislocations, resulting in a formation of high-density dislocation networks for a prominent creep resistance finally^3,19^. On the contrary, alloys with a relatively lower lattice misfit would produce weaker γ/γ′ interfaces, which could not prevent dislocations from cutting the γ′ precipitates and then lead to an occurrence of creeping at the early stage^80^.

In addition, the microstructural evolution of superalloys would vary with the creep conditions, resulting in diverse influences on creep rupture lifetime. In the condition of intermediate temperature/intermediate stress (900 °C/392 MPa), the rafting process of Ni-base SC superalloys with a large lattice misfit is slow, in which the primary creep stage could last up to 200 h approximately^19^. Even after creeping for 200 h, the γ′ morphology still keeps the cuboidal shape and the dislocations are constrained in the horizontal γ channels and have not cut into the γ′ precipitates. However, the primary creep stage is not obvious in some alloys with a relatively small lattice misfit^2^.

While in the condition of high temperature/low stress (1100 °C/137 MPa), the γ′ particles could not keep their cuboidal shape at the primary creep stage, and they would be merged together to form rafted structures (i.e., γ′ lamellae) in superalloys. An irregular rafted structure could be cut and climbed by dislocations, which is detrimental to the rupture lifetime. Lots of existing results have demonstrated that the formation of perfect, continuous, and finer γ′ lamellae is contributed to the prominent creep resistance, which requires a moderate lattice misfit^2,3,75,82,83^. After crept for about 10 h at the primary stage, a rafted lamellar microstructure has been occurred in superalloys, and the formation of high-density dislocation networks could impede dislocations cutting into the γ′ lamellae. Although a large lattice misfit could accelerate the creep rate at the primary stage, it is more beneficial to the formation of perfect continuous γ′ lamellae, which would lead to a minimum creep rate at the secondary creep stage^6,84^.

**Figure 3:**
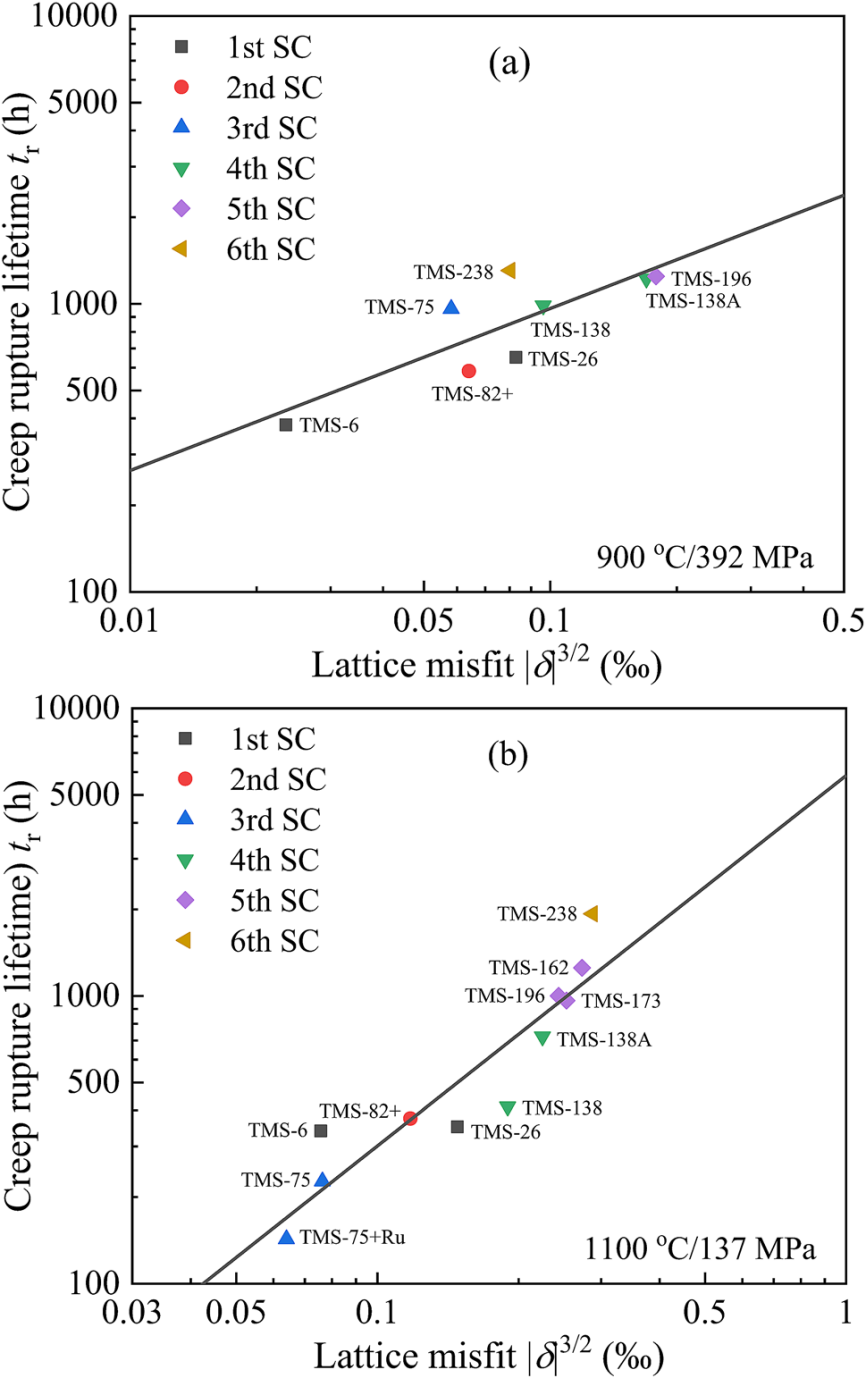
Relationship between the lattice misfit *δ* and creep rupture lifetime *t*_*r*_ of typical TMS series of SC superalloys with prominent creep properties at 900 °C/392 MPa (**a**) and 1100 °C/137 MPa (**b**), respectively.

Generally, the lattice misfit values are limited in the range of 0.2% <|*δ*|< 0.5% for Ni-base SC superalloys to achieve the cuboidal γ′ nanoprecipitates with an appropriate particle size. The more negative lattice misfit could lead to a higher interface strength for impeding the movement of dislocations, which could finally result in a higher creep rupture lifetime. In order to investigate the effect of lattice misfit on the creep rupture lifetime *t*_*r*_ of TMS series of superalloys from the 1st to 6th generation, we applied a double logarithmic function of lg*t*_*r*_–lg|* δ*|^3/2^ to show the relationship between them, as seen in Fig. 3. The parameter of lg*t*_*r*_ is referred from the Larson-Miler equation that represents the creep-resistant property^85^, and the |*δ*|^3/2^ is referred from the coherent strengthening mechanism given in Eq. (2) to represent the yield strength increment^66,67^.

It could be found that the lg*t*_*r*_ at both 900 °C/392 MPa and 1100 °C/137 MPa follows a linear relationship with the lg|*δ*|^3/2^. Moreover, the linear slope (0.56) of lg*t*_*r*_ with lg|*δ*|^3/2^ at 900 °C/392 MPa is less than that (1.29) at 1100 °C/137 MPa, which might be related to the different creep mechanisms. Under the case of 1100 °C/137 MPa, the γ′ nanoprecipitates are rafted rapidly to form the lamella microstructure, in which the integrity of the lamellae and the lamellar spacing, as well as the high-density of dislocation networks are crucial during the creeping process.

### Matching among comprehensive properties of Ni-base superalloys

Besides the excellent HT mechanical properties, the HT oxidation and corrosion of Ni-base superalloys should also be considered due to the harsh service environments. Appropriate composition adjustments of SC superalloys have been carried out not only to their HT mechanical properties but also to the oxidation- and corrosion-resistances. In the 1st generation of TMS SC superalloys, the TMS-277, TMS-285, TMS-286, and TMS-278 are all developed from the base TMS-6 alloy (Table S1). For the first three SC superalloys, the Re element was added into the TMS-6 and a small amount of Nb was substituted for Ta to improve the hot corrosion resistance and to decrease the density of TMS-6 simultaneously^23^. Moreover, a minor amount of Si was also added into the TMS-286 for the improvement of oxidation-resistance since Si can accelerate the formation of continuous and compact Al_2_O_3_ scales^86,87^. For the TMS-278 superalloy, a small amount of Mo element was added into the TMS-6 to enhance the thermal–mechanical fatigue lifetime^23^. However, the additions and adjustments of all these elements in TMS-6 deteriorated indeed the creep rupture lifetime at both 900 °C/392 MPa and 1100 °C/137 MPa, as seen in Table S1. From the viewpoint of the cluster formula compositions, the total atom number of cluster formula of these alloys is almost close to *Z* = 17, in which the total amounts of the $\overline{{{\text{Al}}}}$ and $\overline{{{\text{Cr}}}}$ series of elements in these alloys are almost comparable, being about $G_{{\overline{{{\text{Cr}}}} }}$ = 2.2–2.4 and $N_{{\overline{{{\text{Al}}}} }}$ = 1 + $G_{{\overline{{{\text{Al}}}} }}$ = 1.5–1.7, respectively (Table S1). The main difference exists in the content of each element, which is attributed to the matching of the mechanical property with the oxidation and corrosion properties. This trade-off of comprehensive properties also appeared in the TMS-138A and its derived SC superalloys^24^. The Si element was added into the TMS-138A to obtain the TMS-138A-Cr + Si and TMS-138A-Mo + Si SC alloys by reducing the amount of Cr and Mo, respectively in which the total atom numbers in the cluster formula units of these three alloys are all equal to *Z* = 15.6. On the one hand, the oxidation-resistances of TMS-138A-Cr + Si and TMS-138A-Mo + Si are significantly superior to that of their base alloy TMS-138A at 1100 °C due to the addition of 1.0 at % Si. It could be validated by the fact that the oxide scale of TMS-138A was spalled with a mass loss of 8.7 mg/cm^2^ after 40 cycles in cyclic oxidation tests (1100 °C in air, 1 h /cycle), while the oxidation weight gain of TMS-138A-Cr + Si and TMS-138A-Mo + Si remained about 0.4 mg/cm^2^ without any spalling even after 400 cycles^24^. On the other hand, compared with these two extended Si-containing alloys, the TMS-138A exhibits the longest creep lifetime (*t*_*r*_ = 722 h) at 1100 °C/137 MPa, although the lattice misfit of TMS-138A (*δ* = − 0.37%) is smaller than that (*δ* = − 0.41%) of TMS-138A-Cr + Si at 1100 °C, which results from the precipitation of harmful TCP phase in the extended brand superalloys^24^.

**Figure 4:**
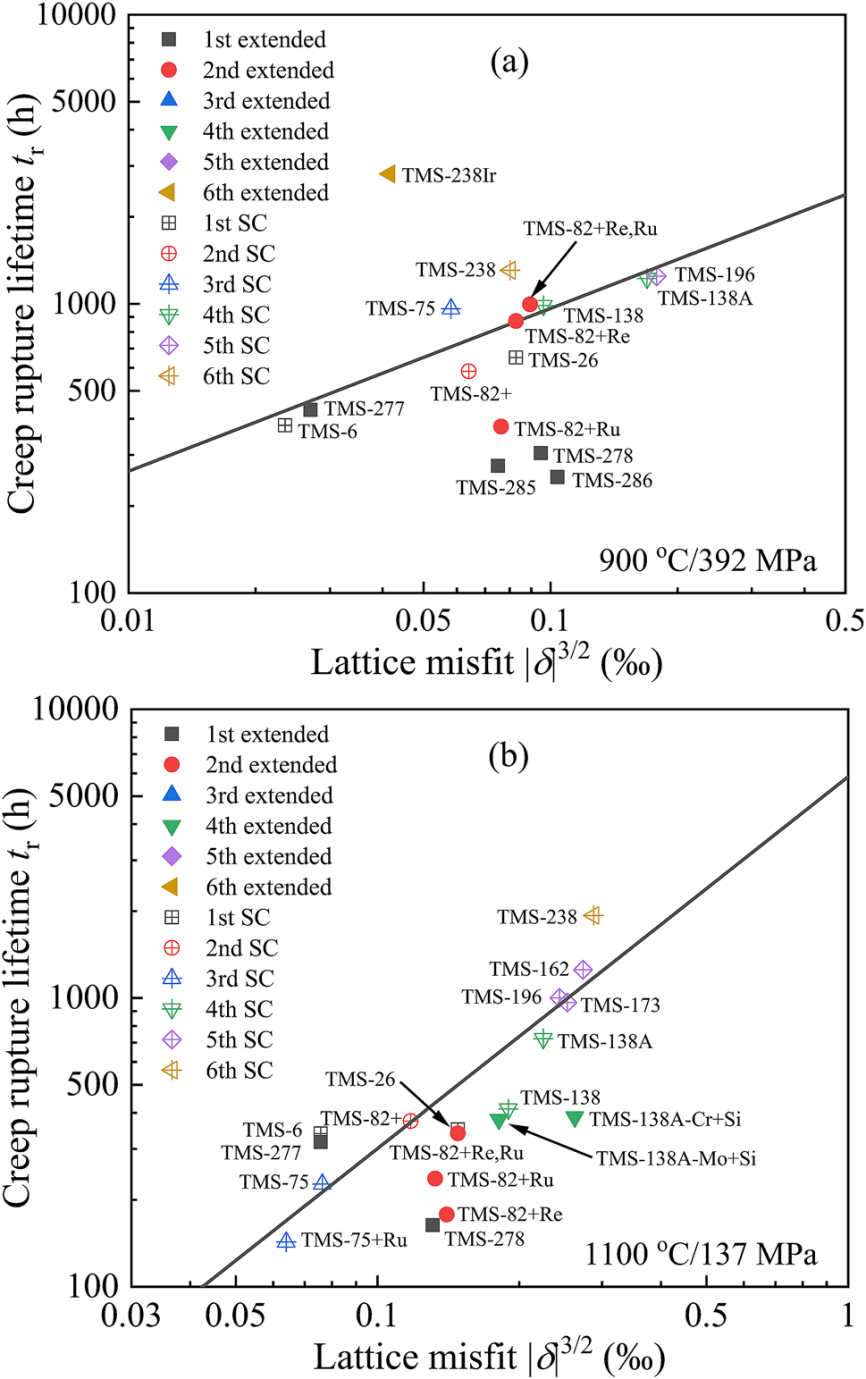
Relationship between the lattice misfit *δ* and creep rupture lifetime *t*_*r*_ of extended brand superalloys with good comprehensive properties at 900 °C/392 MPa (**a**) and 1100 °C/137 MPa (**b**), respectively.

Figure 4 exhibits the lg*t*_*r*_–lg|*δ*|^3/2^ relationship of the extended brand superalloys (solid points) at both 900 °C/392 MPa and 1100 °C/137 MPa based on the linear relationship of typical alloys (hollow points), from which it is found obviously that the improvements of corrosion and oxidation resistances are at the expense of the creep rupture lifetime more or less. One of the main reasons is the precipitation of detrimental TCP phases in alloys due to the addition of several elements of Re, Si, etc.^59,60,88,89^. It has been demonstrated by lots of experiments that a large amount of coarse TCP particles appeared in TMS-286, TMS-278 and TMS-138A-Cr + Si superalloys during the creep process at HTs, compared with TMS-6 and TMS-138A alloys, which would reduce the rupture lifetime drastically. On the contrary, the TMS-238Ir superalloy exhibits a much longer rupture lifetime (*t*_*r*_ = 2813 h) than TMS-238 (*t*_*r*_ = 1306 h) at 900 °C/392 MPa, which is ascribed to the fact that the Ir substitution for Ru can significantly delay the precipitation of TCP phases^56,90^.

## Thoughts on the composition rules of Ni-base SC superalloys

It is emphasized that both the morphology of γ′ nanoprecipitates and the formation of harmful TCP phases are closely related to the chemical composition of Ni-base SC superalloys. Although the existing design methods^14,34,37,39^ could predict the γ/γ′ microstructural stability and the precipitation of TCP phase well, it is difficult to establish the quantitative relationship between alloy composition and HT properties. By analyzing the composition evolution of TMS series of SC superalloys in light of the lattice misfit of γ/γ′ and the creep rupture lifetime, an ideal cluster formula of ${[}\overline{{{\text{Al}}}} {-}\overline{{{\text{Ni}}}} 12{](}\overline{{{\text{Al}}}} 1.5\overline{{{\text{Cr}}}} 1.5{)}$ with the optimum comprehensive property is finally achieved.

**Figure 5:**
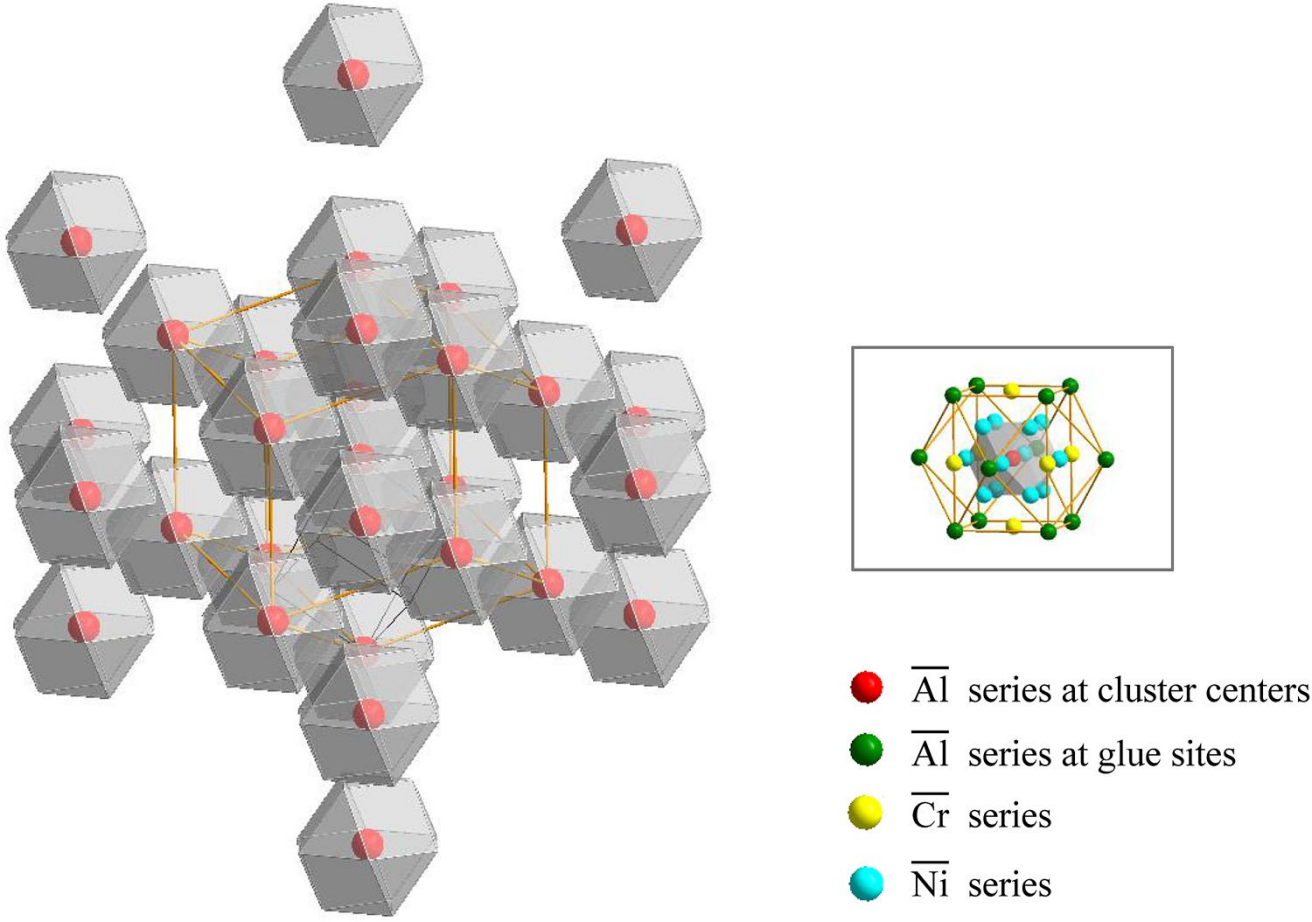
Schematic diagram of a supercluster structural model containing 288 atoms constituted of eighteen basic cluster units of ${[}\overline{{{\text{Al}}}} {-}\overline{{{\text{Ni}}}} 12{](}\overline{{{\text{Al}}}} 1.5\overline{{{\text{Cr}}}} 1.5{)}$, in which each octahedron represents a basic cluster unit containing $\overline{{{\text{Al}}}}$, $\overline{{{\text{Cr}}}}$, and $\overline{{{\text{Ni}}}}$ series in the inset. These basic units are packed according to the cluster model of [CN14 cluster](glue atom)_3_ with *Z* = 18 in body-centered-cubic BCC lattice^43^. The cluster center and shell in the basic cluster unit are occupied by red atom spheres (representing $\overline{{{\text{Al}}}}$ series of elements) and cyan atom spheres ($\overline{{{\text{Ni}}}}$ series), respectively, and the glue sites are co-occupied by green spheres ($\overline{{{\text{Al}}}}$ series) and yellow spheres ($\overline{{{\text{Cr}}}}$ series) with equi-molar ratio, as seen in the distribution of the second nearest-neighbor atoms of the inset.

However, there still exist several issues to be solved in the future work. The present cluster model classifies the alloying elements into three series, $\overline{{{\text{Al}}}}$ series, $\overline{{{\text{Cr}}}}$ series, and $\overline{{{\text{Ni}}}}$ series, but it does not consider how to tune the amount of every specific element in the same series, which is critical to the creep resistance. Therefore, it is necessary to establish a supercluster stacking model that can represent the addition of some trace elements, based on the present cluster structural units with *Z* = 16. One of the potential approaches is that when the cluster structural units of ${[}\overline{{{\text{Al}}}} {-}\overline{{{\text{Ni}}}} 12{](}\overline{{{\text{Al}}}} 1.5\overline{{{\text{Cr}}}} 1.5{)}$ are regarded as a colony and packed in light of the cluster unit of [CN14 cluster](glue atom)_3_ (*Z* = 18) in body-centered-cubic BCC lattice^43^, a regular supercluster model containing 288 atoms (*Z*′ = 288) would be constructed, as seen in Fig. 5, in which the distribution of trace elements could be uncovered obviously in these superclusters units.

Besides, the accurate lattice misfit of γ/γ′ is always obtained from experiments, which should be calculated through some formulas. If the phase compositions of γ and γ′ could be unveiled, the lattice misfit would be obtained easily. So, we can use the cluster structural model to establish the cluster formula of γ and γ′ phase, respectively, and the lattice constants of γ and γ′ will be calculated with the guide of the first-principles calculation. Thus, the lattice misfit of γ/γ′ at different temperatures could also be calculated. Such cluster model is also called the dual-cluster model, in which the different partitions of the two cluster units would present the volume fraction of γ′ precipitates.

Finally, the machine learning method should be introduced into superalloy systems to build a bridge among the alloy composition, microstructure, and property, since there have existed abundant experimental and simulated data up to now. Several existing results have demonstrated that some characteristic parameters in each specific system should be added into the machine learning for precise predictions from composition to property and from property to composition^91,92^. Recently, the cluster formula approach has been embedded into the machine learning to regulate the interactions among alloying elements and the base element in multi-component Ti alloy systems^93^, from which it is found that this method can realize a highly-accurate prediction and design of composition alloys with prominent properties. Therefore, it is believable that the cluster-formula-embedded machine learning method would well predict the relationship between the chemical composition and properties of Ni-base SC superalloys, combined with some characteristic parameters, such as the lattice misfit of γ/γ′, the particle size and volume fraction of γ′ precipitates, etc..

## Conclusions

By exploring the composition rules of TMS series of Ni-base single crystal superalloys with the cluster formula approach and the influence of composition on the creep properties, several key points could be summarized as follows:

(1) The cluster formula of TMS series of superalloys is expressed with the ${[}\overline{{{\text{Al}}}} {-}\overline{{{\text{Ni}}}} 12{](}\overline{{{\text{Al}}}} {, }\overline{{{\text{Cr}}}} {)}m$, in which all the alloying elements are classified into three groups, $\overline{{{\text{Al}}}}$ series, $\overline{{{\text{Cr}}}}$ series, and $\overline{{{\text{Ni}}}}$ series. The total atom number *Z* values of the cluster formula units of these alloys vary from *Z* ~ 17 to *Z* ~ 15.5, and then to *Z* ~ 16 with the alloy development from the 1st to the 6th generation, where the superalloys with prominent creep resistance exhibit an ideal cluster formula of ${[}\overline{{{\text{Al}}}} {-}\overline{{{\text{Ni}}}} 12{](}\overline{{{\text{Al}}}} 1.5\overline{{{\text{Cr}}}} 1.5{)}$ with *Z* = 16. Similar tendency of composition evolution also appears in the PWA and CMSX series of SC superalloys. Based on the ideal cluster formula, it is possible to realize an optimized balance of the HT mechanical property and the oxidation/and corrosion resistances through fine-tuning the amounts of alloying elements in each group.

(2) The relationship between the lattice misfit of γ/γ′ and the creep rupture lifetime of TMS series of SC superalloys is established, showing a linear correlation in the form of lg*t*_*r*_–lg|*δ*|^3/2^ at both conditions of 900 °C/392 MPa and 1100 °C/137 MPa. It would be attributed to the optimization and exploration of new alloy composition with better creep property, combined with cluster formula approach. However, this linear relationship does not appear in those superalloys where the oxidation and corrosion resistances are specially emphasized.

## Methods

Composition analysis of existing Ni-base single crystal superalloys were carried out by the cluster formula approach^40,49^, in which all the alloying elements were classified into three groups, $\overline{{{\text{Al}}}}$ series, $\overline{{{\text{Cr}}}}$ series, and $\overline{{{\text{Ni}}}}$ series. The supercluster structural model containing 288 atoms were constructed by the free VESTA software^94^. The parameter data of these single crystal superalloys, including the lattice misfit *δ* between γ and γ′, and the creep rupture lifetime *t*_*r*_ under both 900 °C/392 MPa and 1100 °C/137 MPa conditions were taken from the literatures and listed in the Supplementary Materials, in which the measurement method could be found elsewhere^6^. Particle size and volume fraction of γ′ of some superalloys used in the present work were also taken from literatures^22,72,74–76^.

## Supplementary information

Supplementary information.

## Data Availability

All data analyzed in this study are included in this published article.
