# Supplementary material for: Composition rules of Ni-base single crystal superalloys and its influence on creep properties via a cluster formula approach
Source: Sci Rep. 2020 Dec 10;10:21621. doi: 10.1038/s41598-020-78690-8 (PMC7728759; doi:10.1038/s41598-020-78690-8)
Supplement: Supplementary file 1 — Supplementary information. [file 41598_2020_78690_MOESM1_ESM.docx]

**Supplementary Materials**

**Composition rules of Ni-base single crystal superalloys and its influence on creep properties via a cluster formula approach**

Chen Chen ^a^, Qing Wang ^a,*^, Chuang Dong ^a,*^, Yu Zhang ^b^, Honggang Dong ^a^

^a^ *Key Laboratory of Materials Modification by Laser, Ion and Electron Beams (Ministry of Education), School of Materials Science and Engineering, Dalian University of Technology, Dalian 116024, China*

^b^ *Department of Basic courses, Liaoning Institute of Science and Technology, Benxi 117004, China*

* Corresponding authors.

*E-mail* addresses: wangq@dlut.edu.cn (Q. Wang), dong@dlut.edu.cn (C. Dong).

**Table S1. Related data of Ni-base single crystal superalloys, including alloy compositions (wt. % and at. %), the cluster formula (the total atom number *Z*, the glue atom number *m*,** **the atom number of** $\overline{\text{Cr}}$ **series (**$\text{G}_{\overline{\text{Cr}}}$**) and** $\overline{\text{Al}}$ **series (**$\text{G}_{\overline{\text{Al}}}$**) in glue sites, and**$\text{ }\text{m}\text{ }\text{=}\text{ }\text{G}_{\bar{\text{Cr}}}\text{ }\text{+}\text{ }\text{G}_{\overline{\text{Al}}}$**), the lattice misfit** ***δ* between γ and γ′, and the creep rupture lifetime *t_r_* at both** **900 ºC/392 MPa and 1100 ºC/137 MPa. The reference number is also listed.**

| **Alloy brands** | | **Alloy compositions** | | **Cluster formula** | ***Z*** | ***m*** | $\text{G}_{\overline{\text{Cr}}}$ | $\text{G}_{\overline{\text{Al}}}$ | **900 ºC** | | **1100 ºC** | | **Ref.** |
| --- | --- | --- | --- | --- | --- | --- | --- | --- | --- | --- | --- | --- | --- |
|  |  | **wt. %** | **at. %** |  |  |  |  |  | ***δ*** **(%)** | ***t_r_* (h) at 392 MPa** | ***δ*** **(%)** | ***t_r_* (h) at 137 MPa** |  |
| 1st | TMS-6 | Ni-5.3Al-9.2Cr-10.4Ta-8.7W | Ni_70.3_Al_12.2_Cr_11.0_Ta_3.6_W_2.9_ | [(Al_0.77_Ta_0.23_)-Ni_12_]  (Al_1.31_Ta_0.38_Cr_1.88_W_0.50_) | 17.07 | 4.07 | 2.38 | 1.69 | -0.082 | 380 | -0.18* | 340 | [1] |
|  | TMS-26 | Ni-5.1Al-8.2Co-5.2Cr-1.9Mo-8.1Ta-11.5W | Ni_64.8_Al_12.0_Co_8.8_Cr_6.3_Mo_1.3_  Ta_2.8_W_4.0_ | [(Al_0.81_Ta_0.19_)-(Ni_10.56_Co_1.44_)]  (Al_1.14_Ta_0.27_Cr_1.03_Mo_0.20_W_0.65_) | 16.30 | 3.30 | 1.88 | 1.42 | -0.19 | 652 | -0.28* | 351 | [2, 3] |
|  | TMS-277 | Ni-5.4Al-9Cr-1.2Nb-1.2Re-10Ta-8W | Ni_69.3_Al_12.5_Cr_10.8_Nb_0.8_Re_0.4_  Ta_3.4_W_2.7_ | [(Al_0.75_Nb_0.05_Ta_0.21_)-(Ni_11.93_Re_0.07_)]  (Al_1.40_Nb_0.09_Ta_0.39_Cr_1.86_W_0.47_) | 17.21 | 4.21 | 2.33 | 1.88 | -0.09 | 429 | -0.18* | 318 | [1] |
|  | TMS-278 | Ni-5.4Al-9Cr-1.2Mo-10Ta-8W | Ni_70.0_Al_12.4_Cr_10.7_Mo_1.3_Ta_3.4_  W_2.7_ | [(Al_0.78_Ta_0.22_)-Ni_12_]  (Al_1.34_Ta_0.37_Cr_1.84_Mo_0.13_W_0.46_) | 17.14 | 4.14 | 2.43 | 1.71 | -0.208 | 304 | -0.26* | 164 | [1] |
|  | TMS-285 | Ni-5.4Al-9Cr-1.2Nb-1.2Re-5.8Ta-8W | Ni_71.6_Al_12.1_Cr_10.5_Nb_0.8_Re_0.4_  Ta_1.9_W_2.6_ | [(Al_0.82_Nb_0.05_Ta_0.13_)-(Ni_11.93_Re_0.07_)]  (Al_1.20_Nb_0.08_Ta_0.19_Cr_1.75_W_0.44_) | 16.66 | 3.66 | 2.19 | 1.47 | -0.178 | 275 |  |  | [1] |
|  | TMS-286 | Ni-5.4Al-9Cr-1.2Nb-1.2Re-0.15Si-5.8Ta-8W | Ni_71.4_Al_12.1_Cr_10.5_Nb_0.8_Re_0.4_  Si_0.3_Ta_1.9_W_2.6_ | [(Al_0.80_Nb_0.05_Si_0.02_Ta_0.13_)-(Ni_11.93_Re_0.07_)]  (Al_1.23_Nb_0.08_Si_0.03_Ta_0.20_Cr_1.75_W_0.44_) | 16.72 | 3.72 | 2.19 | 1.53 | -0.221 | 252 |  |  | [1] |
| 2nd | TMS-82+ | Ni-5.3Al-7.8Co-4.9Cr-0.1Hf-1.9Mo-2.4Re-6Ta-0.5Ti-8.7W | Ni_66.0_Al_12.2_Co_8.2_Cr_5.9_Hf_0.03_  Mo_1.2_Re_0.8_Ta_2.1_Ti_0.6_W_2.9_ | [(Al_0.82_Ta_0.14_Ti_0.04_)-(Ni_10.56_Co_1.31_Re_0.13_)]  (Al_1.13_Ta_0.19_Ti_0.06_Cr_0.94_Mo_0.2_W_0.47_) | 15.99 | 2.99 | 1.60 | 1.38 | -0.16 | 584 | -0.24 | 375 | [3-6] |
|  | TMS-82+Ru | Ni-5.2Al-7.6Co-4.5Cr-0.1Hf-1.8Mo-2.4Re-2Ru-5.9Ta-0.5Ti-8.5W | Ni_65.6_Al_12.1_Co_8.1_Cr_5.4_Hf_0.04_  Mo_1.2_Re_0.8_Ru_1.2_Ta_2.0_Ti_0.7_W_2.9_ | [(Al_0.82_Ta_0.14_Ti_0.04_)-(Ni_10.40_Co_1.28_Re_0.13_Ru_0.20_)]  (Al_1.09_Ta_0.19_Ti_0.06_Cr_0.86_Mo_0.19_W_0.46_) | 15.84 | 2.84 | 1.50 | 1.34 | -0.18 | 376 | -0.26* | 237 | [4] |
|  | TMS-82+Re | Ni-5.3Al-7.6Co-4.5Cr-0.1Hf-1.8Mo-3.5Re-5.9Ta-0.5Ti-8.6W | Ni_65.8_Al_12.3_Co_8.1_Cr_5.9_Hf_0.04_  Mo_1.2_Re_1.2_Ta_2.0_Ti_0.7_W_2.9_ | [(Al_0.82_Ta_0.14_Ti_0.04_)-(Ni_10.52_Co_1.29_Re_0.19_)]  (Al_1.14_Ta_0.19_Ti_0.06_Cr_0.94_Mo_0.19_W_0.47_) | 15.99 | 2.99 | 1.60 | 1.39 | -0.19 | 872 | -0.27* | 178 | [4] |
|  | TMS-82+Re, Ru | Ni-5.2Al-7.6Co-4.5Cr-0.1Hf-1.7Mo-3.5Re-2Ru-5.8Ta-0.5Ti-8.3W | Ni_65.2_Al_12.1_Co_8.1_Cr_5.4_Hf_0.04_  Mo_1.1_Re_1.2_Ru_1.2_Ta_2.0_Ti_0.7_W_2.8_ | [(Al_0.82_Ta_0.14_Ti_0.04_)-(Ni_10.33_Co_1.29_Re_0.13_Ru_0.20_)]  (Al_1.10_Ta_0.18_Ti_0.06_Cr_0.86_Mo_0.18_W_0.45_) | 15.84 | 2.84 | 1.49 | 1.35 | -0.2 | 995 | -0.28* | 340 | [4] |
| 3rd | TMS-75 | Ni-6Al-12Co-3Cr-0.1Hf-2Mo-5Re-6Ta-6W | Ni_63.1_Al_13.7_Co_12.6_Cr_3.6_Hf_0.03_  Mo_1.3_Re_1.7_Ta_2.0_W_2.0_ | [(Al_0.87_Ta_0.13_)-(Ni_9.79_Co_1.95_Re_0.26_)]  (Al_1.26_Ta_0.19_Cr_0.55_Mo_0.20_W_0.31_) | 15.52 | 2.52 | 1.07 | 1.45 | -0.15 | 961 | -0.18 | 227 | [3, 7-10] |
|  | TMS-75(+Ru) | Ni-6Al-12Co-3Cr-0.1Hf-2Mo-5Re-1.6Ru-6Ta-6W | Ni_61.8_Al_13.8_Co_12.7_Cr_3.6_Hf_0.03_  Mo_1.3_Re_1.7_Ru_1.0_Ta_2.1_W_2.0_ | [(Al_0.87_Ta_0.13_)-(Ni_9.62_Co_1.97_Re_0.26_Ru_0.15_)]  (Al_1.28_Ta_0.19_Cr_0.56_Mo_0.2_W_0.32_) | 15.55 | 2.55 | 1.08 | 1.47 | -0.13 |  | -0.16 | 143 | [11] |
| 4th | TMS-138 | Ni-5.9Al-5.8Co-3.2Cr-0.1Hf-2.8Mo-5Re-2Ru-5.6Ta-5.9W | Ni_67.7_Al_13.6_Co_6.1_Cr_3.8_Hf_0.03_  Mo_1.8_Re_1.7_Ru_1.2_Ta_1.9_W_2.0_ | [(Al_0.88_Ta_0.12_)-(Ni_10.59_Co_0.96_Re_0.26_Ru_0.19_)]  (Al_1.26_Ta_0.18_Cr_0.60_Mo_0.28_W_0.31_) | 15.63 | 2.63 | 1.20 | 1.43 | -0.21 | 987 | -0.33 | 412 | [10,12,13] |
|  | TMS-138A | Ni-5.7Al-5.8Co-3.2Cr-0.1Hf-2.9Mo-5.8Re-3.6Ru-5.6Ta-5.6W | Ni_66.5_Al_13.4_Co_6.2_Cr_3.9_Hf_0.04_  Mo_1.9_Re_2.0_Ru_2.3_Ta_2.0_W_1.9_ | [(Al_0.87_Ta_0.13_)-(Ni_10.37_Co_0.97_Re_0.31_Ru_0.35_)]  (Al_1.21_Ta_0.18_Cr_0.61_Mo_0.30_W_0.30_) | 15.60 | 2.60 | 1.21 | 1.39 | -0.306 | 1224 | -0.37 | 722 | [14] |
|  | TMS-138A-Cr+Si | Ni-5.7Al-5.8Co-2.4Cr-0.1Hf-2.9Mo-5.9Re-3.7Ru-0.45Si-5.7Ta-5.5W | Ni_66.4_Al_13.4_Co_6.2_Cr_2.9_Hf_0.04_  Mo_1.9_Re_2.0_Ru_2.3_Si_1.0_Ta_2.0_W_1.9_ | [(Al_0.82_Si_0.06_Ta_0.12_)-(Ni_10.36_Co_0.97_Re_0.31_Ru_0.36_)]  (Al_1.26_Si_0.10_Ta_0.19_Cr_0.45_Mo_0.30_W_0.29_) | 15.59 | 2.59 | 1.05 | 1.54 |  |  | -0.41 | 387 | [14] |
|  | TMS-138A-Mo+Si | Ni-5.7Al-5.8Co-3.2Cr-0.1Hf-1.4Mo-5.9Re-3.7Ru-0.45Si-5.8Ta-5.6W | Ni_66.4_Al_13.4_Co_6.2_Cr_3.9_Hf_0.04_  Mo_0.9_Re_2.0_Ru_2.3_Si_1.0_Ta_2.0_W_1.9_ | [(Al_0.81_Si_0.06_Ta_0.12_)-(Ni_10.37_Co_0.96_Re_0.31_Ru_0.36_)]  (Al_1.25_Si_0.09_Ta_0.19_Cr_0.60_Mo_0.14_W_0.30_) | 15.57 | 2.57 | 1.04 | 1.53 |  |  | -0.32 | 380 | [14] |
| 5th | TMS-162 | Ni-5.8Al-5.8Co-2.9Cr-0.1Hf-3.9Mo-4.9Re-6Ru-5.6Ta-5.8W | Ni_64.4_Al_13.7_Co_6.3_Cr_3.6_Hf_0.04_  Mo_2.6_Re_1.7_Ru_3.8_Ta_2.0_W_2.0_ | [(Al_0.87_Ta_0.13_)-(Ni_10.15_Co_0.99_Re_0.26_Ru_0.60_)]  (Al_1.29_Ta_0.19_Cr_0.56_Mo_0.41_W_0.32_) | 15.76 | 2.76 | 1.29 | 1.47 |  |  | -0.42* | 1252 | [15-17] |
|  | TMS-173 | Ni-5.6Al-5.6Co-2.8Cr-0.1Hf-2.8Mo-6.9Re-5Ru-5.6Ta-5.6W | Ni_65.7_Al_13.3_Co_6.1_Cr_3.5_Hf_0.04_  Mo_1.9_Re_2.4_Ru_3.2_Ta_2.0_W_2.0_ | [(Al_0.87_Ta_0.13_)-(Ni_10.19_Co_0.95_Re_0.37_Ru_0.49_)]  (Al_1.20_Ta_0.18_Cr_0.54_Mo_0.29_W_0.30_) | 15.51 | 2.51 | 1.13 | 1.38 |  |  | -0.4* | 964 | [18,19] |
|  | TMS-196 | Ni-5.6Al-5.6Co-4.6Cr-0.1Hf-2.4Mo-6.4Re-5Ru-5.6Ta-5W | Ni_64.6_Al_13.2_Co_6.0_Cr_5.6_Hf_0.04_  Mo_1.6_Re_2.2_Ru_3.1_Ta_2.0_W_1.7_ | [(Al_0.87_Ta_0.13_)-(Ni_10.21_Co_0.95_Re_0.34_Ru_0.50_)]  (Al_1.21_Ta_0.18_Cr_0.89_Mo_0.25_W_0.27_) | 15.80 | 2.80 | 1.41 | 1.39 | -0.317 | 1245 | -0.39 | 1001 | [18,20,21] |
| 6th | TMS-238 | Ni-5.9Al-6.5Co-4.6Cr-0.1Hf-1.1Mo-6.4Re-5Ru-7.6Ta-4W | Ni_63.5_Al_13.8_Co_7.0_Cr_5.6_Hf_0.04_  Mo_0.7_Re_2.2_Ru_3.1_Ta_2.7_W_1.4_ | [(Al_0.84_Ta_0.16_)-(Ni_10.05_Co_1.11_Re_0.34_Ru_0.50_)]  (Al_1.36_Ta_0.26_Cr_0.89_Mo_0.12_W_0.22_) | 15.84 | 2.84 | 1.22 | 1.62 | -0.186 | 1306 | -0.44 | 1930 | [22-26] |
|  | TMS-238Ir | Ni-5.9Al-6.2Co-4.4Cr-0.1Hf-1.1Mo-6.1Re-7.3Ta-3.8W-9.1Ir | Ni_63.4_Al_13.9_Co_7.0_Cr_5.6_Hf_0.04_  Mo_0.7_Re_2.2_Ta_2.7_W_1.4_Ir_3.1_ | [(Al_0.84_Ta_0.16_)-(Ni_10.05_Co_1.11_Re_0.35_Ir_0.50_)]  (Al_1.36_Ta_0.26_Cr_0.89_Mo_0.12_W_0.22_) | 15.84 | 2.84 | 1.22 | 1.62 | -0.12 | 2813 |  |  | [26, 27] |
| 1st | CMSX-2 | Ni-5.5Al-5.0Co-8Cr-0.6Mo-6Ta-1Ti-8W | Ni_67.3_Al_12.2_Co_5.1_Cr_9.2_Mo_0.4_  Ta_2.0_Ti_1.3_W_2.6_ | [(Al_0.79_Ta_0.13_Ti_0.08_)-(Ni_11.16_Co_0.84_)]  (Al_1.24_Ta_0.20_Ti_0.13_Cr_1.53_Mo_0.06_W_0.43_) | 16.59 | 3.59 | 2.02 | 1.56 |  |  |  |  | [28] |
|  | CMSX-3 | Ni-5.6Al-4.6Co-8Cr-0.1Hf-0.6Mo-6Ta-1Ti-8W | Ni_67.4_Al_12.4_Co_4.7_Cr_9.2_Hf_0.03_  Mo_0.4_Ta_2.0_Ti_1.3_W_2.6_ | [(Al_0.79_Ta_0.13_Ti_0.08_)-(Ni_11.22_Co_0.78_)]  (Al_1.27_Ta_0.20_Ti_0.13_Cr_1.53_Mo_0.06_W_0.43_) | 16.64 | 3.64 | 2.03 | 1.61 |  |  |  |  | [29] |
|  | CMSX-6 | Ni-4.8Al-5Co-9.8Cr-0.1Hf-3Mo-2Ta-4.7Ti | Ni_67.0_Al_9.9_Co_4.7_Cr_10.5_Hf_0.03_  Mo_1.7_Ta_0.6_Ti_5.5_ | [(Al_0.62_Ta_0.04_Ti_0.34_)-(Ni_11.21_Co_0.79_)]  (Al_1.04_Ta_0.06_Ti_0.57_Cr_1.76_Mo_0.29_) | 16.72 | 3.72 | 2.05 | 1.68 |  |  |  |  | [30] |
| 2nd | CMSX-4 | Ni-5.6Al-9Co-6.5Cr-0.1Hf-0.6Mo-3Re-6.5Ta-1Ti-6W | Ni_63.8_Al_12.6_Co_9.3_Cr_7.6_Hf_0.03_  Mo_0.4_Re_1.0_Ta_2.2_Ti_1.3_W_2.0_ | [(Al_0.79_Ta_0.14_Ti_0.08_)-(Ni_10.34_Co_1.50_Re_0.16_)]  (Al_1.26_Ta_0.22_Ti_0.13_Cr_1.23_Mo_0.06_W_0.32_) | 16.21 | 3.21 | 1.61 | 1.60 |  | 499 | -0.13 | 136 | [31,32] |
|  | CMSX-4plus | Ni-5.7Al-10Co-3.5Cr-0.1Hf-0.6Mo-4.8Re-8Ta-0.85Ti-6W | Ni_64.2_Al_13.2_Co_10.6_Cr_4.2_Hf_0.03_  Mo_0.4_Re_1.6_Ta_2.8_Ti_1.1_W_2.0_ | [(Al_0.77_Ta_0.16_Ti_0.06_)-(Ni_10.09_Co_1.66_Re_0.25_)]  (Al_1.30_Ta_0.27_Ti_0.11_Cr_0.66_Mo_0.06_W_0.32_) | 15.72 | 2.72 | 1.04 | 1.68 |  |  |  |  | [33] |
| 3rd | CMSX-10 | Ni-5.7Al-3Co-2Cr-0.03Hf-0.4Mo-0.1Nb-6Re-8Ta-0.2Ti-5W | Ni_74.1_Al_13.2_Co_3.2_Cr_2.4_Hf_0.01_  Mo_0.3_Nb_0.1_Re_2.0_Ta_2.8_Ti_0.3_W_1.7_ | [(Al_0.81_Nb_0.00_Ta_0.17_Ti_0.02_)-(Ni_11.21_Co_0.48_Re_0.30_)]  (Al_1.19_Nb_0.01_Ta_0.25_Ti_0.02_Cr_0.36_Mo_0.04_  W_0.26_) | 15.13 | 2.13 | 0.66 | 1.47 |  |  |  | 330 | [34,35] |
|  | CMSX-10K | Ni-5.8Al-3.3Co-2.3Cr-0.4Mo-0.1Nb-6.3Re-8.3Ta-0.2Ti-5.5W | Ni_72.7_Al_13.5_Co_3.5_Cr_2.8_Mo_0.3_  Nb_0.1_Re_2.1_Ta_2.9_Ti_0.3_W_1.9_ | [(Al_0.81_Nb_0.00_Ta_0.17_Ti_0.02_)-(Ni_11.13_Co_0.54_Re_0.33_)]  (Al_1.26_Nb_0.01_Ta_0.27_Ti_0.02_Cr_0.43_Mo_0.04_  W_0.29_) | 15.32 | 2.32 | 0.75 | 1.56 |  |  |  | 333 | [1] |
| 1st | PWA1480 | Ni-5Al-5Co-10Cr-12Ta-1.5Ti-4W | Ni_64.7_Al_11.3_Co_5.2_Cr_11.7_Ta_4.0_  Ti_1.9_W_1.3_ | [(Al_0.65_Ta_0.23_Ti_0.11_)-(Ni_11.11_Co_0.89_)]  (Al_1.28_Ta_0.46_Ti_0.22_Cr_2.01_W_0.23_) | 17.19 | 4.19 | 2.23 | 1.95 |  | 82.6 |  | 14 | [5,36] |
|  | PWA1483 | Ni-3.6Al-9Co-12.8Cr-1.9Mo-4Ta-4Ti-3.8W | Ni_60.5_Al_7.8_Co_8.9_Cr_14.3_Mo_1.2_  Ta_1.3_Ti_4.9_W_1.2_ | [(Al_0.56_Ta_0.09_Ti_0.35_)-(Ni_10.46_Co_1.54_)]  (Al_0.79_Ta_0.13_Ti_0.49_Cr_2.48_Mo_0.20_W_0.21_) | 17.30 | 4.30 | 2.89 | 1.41 |  |  |  |  | [37] |
| 2nd | PWA1484 | Ni-5.6Al-10Co-5Cr-0.1Hf-2Mo-3Re-8.7Ta-6W | Ni_63.2_Al_12.9_Co_10.6_Cr_6.0_Hf_0.03_  Mo_1.3_Re_1.0_Ta_3.0_W_2.0_ | [(Al_0.81_Ta_0.19_)-(Ni_10.14_Co_1.70_Re_0.16_)]  (Al_1.26_Ta_0.29_Cr_0.96_Mo_0.21_W_0.33_) | 16.05 | 3.05 | 1.49 | 1.55 |  |  |  | 139 | [38,39] |
|  | PWA1487 | Ni-5.6Al-10Co-5Cr-0.25Hf-1.9Mo-3Re-8.4Ta-5.9W | Ni_63.4_Al_12.9_Co_10.5_Cr_6.0_Hf_0.09_  Mo_1.2_Re_1.0_Ta_2.9_W_2.0_ | [(Al_0.82_Ta_0.18_)-(Ni_10.15_Co_1.69_Re_0.16_)]  (Al_1.25_Ta_0.28_Cr_0.96_Mo_0.20_W_0.32_) | 16.00 | 3.00 | 1.47 | 1.52 |  |  |  |  | [40] |
| 4th | MX-4  / PWA1497 | Ni-5.6Al-16.5Co-2Cr-0.15Hf-2Mo-6Re-3Ru-8.3Ta-6W | Ni_55.5_Al_13.4_Co_18.1_Cr_2.5_Hf_0.05_  Mo_1.3_Re_2.1_Ru_1.9_Ta_3.0_W_2.1_ | [(Al_0.82_Ta_0.18_)-(Ni_8.59_Co_2.80_Re_0.32_Ru_0.30_)]  (Al_1.25_Ta_0.28_Cr_0.38_Mo_0.21_W_0.33_) | 15.45 | 2.45 | 0.92 | 1.53 |  |  |  | 141 | [23,38] |

* The lattice misfit *δ* values at 1100 ºC were calculated by JmatPro software.

It is also noted that the lattice misfit at 900 ºC were all calculated by the NIMS in-house alloy design program (NIMS-ADP), and those at 1100 ºC were mainly obtained by the high-temperature XRD measurements.

**References:**

1. Kawagishi, K. *et al.* Development of low or zero‐rhenium high‐performance Ni‐base single crystal superalloys for jet engine and power generation applications. *Superalloys 2016*, 115-122 (2016).
2. Kobayashi, T. *et al.* Development of 4th generation SC superalloys without Re. *J. Jpn. Inst. Met.* **69**, 272-275 (2005).
3. Osawa, M., Shiraishi, H., Yokokawa, T., Harada, H., & Kobayashi, T. 3D-FEM calculations of rafting in Ni-base superalloys based on high temperature elastic and lattice parameters. *Superalloy*, 977-985 (2004).
4. Yokokawa, T., Koizumi, Y., Kobayashi, T. & Harada, H. Effect of Re and Ru additions to second generation nickel-base single crystal superalloy TMS-82+. *J. Jpn. Inst. Met.* **70**, 670-673 (2006).
5. Kobayashi, T., Harada, H., Osawa, M. & Sato, A. Creep strength of Co-free Ni-base single crystal superalloys. *J. Jpn. Inst. Met.* **69**, 707-710 (2005).
6. Suzuki, T. *et al.* Effect of alloying elements for designing of advanced co-free ni-base superalloys. *J. Jpn. Inst. Met.* **72**, 8-11 (2008).
7. Koizumi, Y. *et al.* Third generation single crystal superalloys with excellent processability and phase stability. *Proc. of 6th Liège conference, Part II, Belgium.* 1089-1098 (1998).
8. Kobayashi, T. *et al.* Development of a third generation DS superalloy. *Superalloy*, 323-328 (2000).
9. Murakumo, T., Kobayashi, T., Koizumi, Y. & Harada, H. Creep behaviour of Ni-base single-crystal superalloys with various γ′ volume fraction. *Acta Materialia* **52**, 3737-3744 (2004).
10. Zhang, J. X., Murakumo, T., Harada, H., Koizumi, Y. & Kobayashi, T. Creep deformation mechanisms in some modern single-crystal superalloys. *Superalloy*, 289-195 (2004).
11. Zhang, J. X., Harada, H., Koizumi, Y. & Kobayashi, T. Dislocation motion in the early stages of high-temperature low-stress creep in a single-crystal superalloy with a small lattice misfit. *Journal of Materials Science* **45**, 523-532 (2010).
12. Koizumi, Y. *et al.* Effects of alloying additions on the creep strength of a fourth generation single-crystal superalloy. *J. Jpn. Inst. Met.* **68**, 206-209 (2004).
13. Yokokawa, T., Harada, H., Kawagishi, K., Koizumi, Y. & Kobayashi, T. Quantitative analysis of creep strengthening factors factors in Ni-base single crystal superalloys. *Superalloy*, 285-292 (2012).
14. Yeh, A. C. *et al.* Development of Si-bearing 4th generation Ni-base single crystal superalloys. *Superalloy*, 619-628 (2008).
15. Koizumi, Y. *et al.* Development of next generation Ni-base single crystal superalloys containing ruthenium. *J. Jpn. Inst. Met.* **67**, 468-471 (2003).
16. Koizumi, Y. *et al.* Development of next-generation Ni-base single crystal superalloys. *Superalloy*, 35-43 (2004).
17. Zhang, J. X., Murakumo, T., Koizumi, Y., Kobayashi, T. & Harada, H. Strengthening by γ/γ′ interfacial dislocation networks in TMS-162—toward a fifth-generation single-crystal superalloy. *Metallurgical and Materials Transactions A* **35**, 1911-1914 (2004).
18. Sato, A. *et al.* A 5th generation Ni-base single crystal superalloy designed for the combination of excellent oxidation resistance and creep strength at elevated temperatures. *J. Jpn. Inst. Met.* **70**, 196-199 (2006).
19. Kawagishi, K. *et al.* Oxidation resistant Ru containing Ni base single crystal superalloys. *Materials Science and Technology* **25**, 271-275 (2009).
20. Yeh, A. C., Sato, A., Kobayashi, T. & Harada, H. On the creep and phase stability of advanced Ni-base single crystal superalloys. *Materials Science and Engineering: A* **490**, 445-451 (2008).
21. Sato, A. *et al.* A 5(th) generation SC superalloy with balanced high temperature properties and processability. *Superalloy*, 131-138 (2008).
22. Takebe, Y. *et al.* Effect of Ir on the Microstructural Stability of the 6th Generation Ni-Base Single Crystal Superalloy, TMS-238. *Journal of the Japan Institute of Metals and Materials* **79**, 227-231 (2015).
23. Kawagishi, K. *et al.* Development of an oxidation-resistant high-strength sixth-generation single-crystal superalloy TMS-238. *Superalloy*, 9-13(2012).
24. Yokokawa, T. *et al.* Design of next generation Ni‐base single crystal superalloys containing Ir: towards 1150°C temperature capability. *Superalloys 2016*, 123-130 (2016).
25. Yuan, Y. *et al.* Creep deformation of a sixth generation Ni-base single crystal superalloy at 800°C. *Materials Science and Engineering: A* **608**, 95-100 (2014).
26. Mori, Y. Creep deformation behavior of Ni-base single crystal superalloys containing Ir. (PhD Dissertation, Waseda University, 2017).
27. Mori, Y. *et al.* Microstructure and creep strength of the Ni-base single crystal superalloys containing Ir substituting for Ru. *Proc. 9th Pacific Rim International Conference on Advanced Materials and Processing.* 765-769 (2016).
28. Caron, P. & Khan, T. Improvement of Creep strength in a nickel-base single-crystal superalloy by heat treatment. *Materials Science and Engineering* **61**, 173-184 (1983).
29. Pollock, T. M. & Argon, A. S. Creep resistance of CMSX-3 nickel base superalloy single crystals. *Acta Metallurgica et Materialia* **40**, 1-30 (1992).
30. Kamaraj, M., Mayr, C., Kolbe, M. & Eggeler, G. On the influence of stress state on rafting in the single crystal superalloy CMSX-6 under conditions of high temperature and low stress creep. *Scripta Materialia* **38**, 589-594 (1998).
31. Bullough, C., Toulios, M., Oehl, M. & Lukas, P. The characterization of the single crystal superalloy CMSX-4 for industrial gas turbine blading applications. *Proc. 6 Liege Conference on Materials for Advanced Power Engineering, Liege.* 5-7 (1998).
32. Hino, T., Yoshioka, Y., Koizumi, Y., Kobayashi, T. & Harada, H. Development and evaluation of high strength Ni-base single crystal superalloy, TMS-82+. *Proc. of the International Gas Turbine Congress*, (2003).
33. Wahl, J. B. & Harris, K. CMSX‐4® plus single crystal alloy development, characterization and application development. *Superalloys 2016*, 25-33 (2016).
34. Link, T., Epishin, A. & Fedelich, B. Inhomogeneity of misfit stresses in nickel-base superalloys: Effect on propagation of matrix dislocation loops. *Philosophical Magazine* **89**, 1141-1159 (2009).
35. Erickson, G. The development and application of CMSX-10. *Superalloys 1996*, 35-44 (1996).
36. Tewari, S. N., Vijayakumar, M., Lee, J. E. & Curreri, P. A. Solutal partition coefficients in nickel-based superalloy PWA-1480. *Materials Science and Engineering: A* **141**, 97-102 (1991).
37. Lamm, M. & Singer, R. F. The effect of casting conditions on the high-cycle fatigue properties of the single-crystal nickel-base superalloy PWA 1483. *Metallurgical and Materials Transactions A* **38**, 1177-1183 (2007).
38. Celel, A. & Duhl, D. Second-generation nickel-base single crystal superalloy. *Superalloys 1988*, 235-244 (1988).
39. Zietara, M., Cetel, A. & Czyrska-Filemonowicz, A. Microstructure stability of 4th generation single crystal superalloy, PWA 1497, during high temperature creep deformation. *Mater. Trans.* **52**, 336-339 (2011).
40. Reed, R. C. *The superalloys: fundamentals and applications*. (Cambridge university press, 2008).
